# Implementation and product- and process evaluation of a co-created gender-informed and culturally-sensitive toolkit to improve symptom recognition and care seeking for ischemic heart disease: RE-AIM framework

**DOI:** 10.1371/journal.pone.0344093

**Published:** 2026-03-05

**Authors:** Bryn Hummel, Dinah L. van Schalkwijk, Nina Zipfel, Pearl T. C. Tuithof, Paula M. C. Mommersteeg, Ralf E. Harskamp, Irene G. M. van Valkengoed

**Affiliations:** 1 Department of Public and Occupational Health, Amsterdam UMC, Location AMC, Amsterdam, the Netherlands; 2 Center of Research on Psychological Disorders and Somatic diseases (CoRPS), Department of Medical and Clinical Psychology, Tilburg University, Tilburg, the Netherlands; 3 Department of General Practice, Amsterdam UMC, Location AMC, Amsterdam, the Netherlands; Tribhuvan University Institute of Medicine, NEPAL

## Abstract

**Background:**

Tailored health messaging can be used to improve symptom recognition of ischemic heart disease (IHD) and reduce barriers to care. Therefore, we propose that a gender-informed and culturally-sensitive toolkit for health messaging could improve health outcomes by promoting adequate care seeking in the community. Hence, we will develop, implement, and evaluate a toolkit to improve IHD-related symptom recognition and care seeking in an ethnically-diverse population.

**Methods:**

We developed a toolkit in co-creation with patients, citizens, community leaders, and healthcare professionals. Subsequently, we pilot-implemented the toolkit through organizing information sessions in four different community-based settings. Finally, using a mixed-methods design, we evaluated the Reach, Effectiveness, Adoption, Implementation, and Maintenance of the toolkit (RE-AIM), including its effect on knowledge gain on symptom recognition and care seeking,.

**Results:**

Approximately 240 people attended the information sessions, of whom 69% women, and the mean age was 63 (standard deviation 10.7) years old. Our results showed a small increase [varying from 53.4–74.4% pre-test to 67.6–85.4% post-test] in knowledge about IHD symptoms. A great willingness to adoption the intervention was observed among the targeted community leaders, and despite several points of improvement, including fidelity to the implementation, attendees and community leaders reflected positively on the toolkit. The main challenge regarding the maintenance of the toolkit was a lack of experience in organizing community events by potential maintenance organizations.

**Conclusion:**

While the co-created toolkit was well-received, and findings concerning the reach, adoption, and implementation were positive, the (cost)effectiveness should be further evaluated to study the long-term impact of the intervention. Moreover, the need for integration of the intervention in current infrastructure constituted a challenge to the maintenance of the toolkit.

## Background

Delays in diagnosis and treatment inherently occur when people do not seek timely care when experiencing symptoms [1]. In particular during acute cardiovascular events, and to a lesser degree in case of chronic cardiovascular conditions, timely care is key to improve medical outcomes and reduce the medical, financial and social burden of ischemic heart disease (IHD) [2]. This is relevant, as IHD is a leading cause of mortality worldwide [3,4] as well as impaired quality of life, and poses a significant (financial) burden on healthcare systems [5,6] and society [7].

Beyond the unequal burden of IHD across population subgroups – including sex and ethnic disparities in IHD [8–13] – studies also show disparities in the interval between symptom onset and care seeking between women and men, and people of different ethnic groups [14–16]. While causes of delays in care seeking are diverse and multifactorial, poor symptom recognition is a main factor contributing to delayed care seeking across population subgroups [16,17], and studies suggest symptom recognition is lower in women and people from ethnic minority groups [16].

In light of the poor IHD symptom recognition, the European Society of Cardiology recommends tailored public health messaging and community-based education, utilizing culturally appropriate language and images to resonate with the target populations, in order to effectively engage ethnic minority groups and inform them on IHD symptoms [15]. To our knowledge, no culturally-sensitive health communication tool to improve IHD symptom recognition and promote adequate care seeking exists in the Netherlands [18].

Therefore, we aimed to develop and pilot-implement a gender-informed and culturally-sensitive toolkit to improve IHD symptom recognition and care seeking in women and men of different ethnic groups in the Netherlands, and evaluate this via a product- and process evaluation. This toolkit is based on earlier qualitative work on care seeking [17] and health information-seeking behavior [19], and was developed in co-creation with people from different communities, to ensure that the product was tailored to the needs, preferences, and experiences of women and men with diverse ethnic backgrounds. The evaluation was conducted using the RE-AIM framework, by evaluating the Reach, Effectiveness, Adoption, Implementation, and Maintenance of interventions [20].

## Materials and methods

In the methods, we start by briefly outlining the development of the intervention components, followed by the implementation strategies and implementation settings. Finally, we describe the strategies used in the product- and process evaluation. We will use the RE-AIM framework for the structure of the paper [20].

### Intervention components

The initial idea for this intervention was developed based on earlier work on care seeking [17] and health information-seeking behavior [19], in which we identified reasons for delays in care, and what people needed to timely seek care. For detailed background information and the rationale behind the intervention components, please refer to the original publications [17,19], S1 File (a brief description of the intervention development) and S2 File (the logic model). In brief, these studies describe that women and men from ethnic minority groups preferred receiving information about IHD via culturally-sensitive, live, interactive information sessions organized in community settings. This toolkit was also designed to be gender-informed, by including information on sex differences in symptoms and risk factors, discussing gendered barriers to care, and gender-responsive by taking into account sex-specific preferences for information sessions, e.g., separating women and men. We hypothesize that knowledge on IHD symptoms and when to seek care will improve IHD symptom recognition, and thereby, improve care seeking for potential IHD symptoms. We use the RE-AIM framework to study the toolkit’s reach, effectiveness, adoption, implementation, and maintenance in a real-world setting. Table 1 provides an overview of the tools in this toolkit, which were used to organize the live information sessions. We subsequently evaluated the Reach, Effectiveness, and Adoption of the live information sessions, and the Implementation and Maintenance of the toolkit, described in more detail below.

**Table 1:** Tools provided in the toolkit.

Purpose	Tool	Short description
Organization and preparation materials	Approaching community leaders	As community leaders were deemed essential for reaching and informing ethnically-diverse communities, this tool offers practical tips for approaching community leaders and building relationships for collaboration.
	Persona’s	These fictional profiles of people from different ethnic backgrounds are based on our qualitative studies. It provides the toolkit user with information about the experiences, needs, behavior, religion, vision on health and sickness, and the main barriers to care of targeted communities (specifically Ghanaian, Afro-Surinamese, South-Asian Surinamese, Turkish, Moroccan, Dutch).
	Live sessions	This tool helps organize a live session by describing a step-by-step approach to the arrangements that need to be made and other aspects that need to be taken into consideration.
	Checklist	This checklist helps organizers check whether they have all the necessities and have executed all the activities needed to host a live session.
	Promotion material	The promotional materials can be used to promote the live information session with communities and are available in 4 languages (Dutch, English, Turkish, Arabic). These materials can be used and adapted by the organization.
Content for educational material	Education plan for educators	The education plan can be used to prepare and structure the presentation. It names the learning goals, provides culturally-sensitive anecdotes that can be used as appealing examples of patients, and names the most important information.
	Dealing with barriers to care	This tool contains a list of all barriers to care identified in our qualitative study and provides possible solutions or ways to address these barriers.
	Preparing for a consultation	This tool provides patients with information and tips about how to prepare for a doctor’s consultation.
	Script for a theater play	This script can be used to create a theater play, which communities can adapt to their own language and culture, and then organize within their communities.
	Slides for the session	These are slides that were developed in collaboration with medical doctors and can be used during the information sessions. The slides are on B1 level, and are available in different languages (Dutch, English, Turkish, and Arabic). The presentation includes several interactive components, including a narrative, an anecdotal story of a patient, and a group discussion of barriers to care. It is important that the medical information provided in these slides is not altered.
	Educational videos	These videos, available in Dutch, English, Turkish, Arabic, Moroccan-Arabic (Darija), and Tamazight, give information about IHD symptoms and when to seek (non)emergency care.
	Information flyer	These flyers, available in Dutch, English, Turkish, and Arabic, give information about IHD symptoms and when to seek (non)emergency care. These can be handed out after the information session to take home and serve as a reminder.

IHD, Ischemic Heart Disease.

### Implementation strategies and settings

During the pilot-implementation study, which took place in May of 2024, the goal was to implement the toolkit by organizing information sessions in four different community settings: a primarily South-Asian Surinamese Mosque, a primarily West-African church, a Turkish mosque, and a physical location rented by South-Asian Surinamese foundation. We aimed to implement the intervention in several different settings representing large ethnic groups in the Netherlands, as this diversity strengthens the external validity. Due to financial and time constraints, we were restricted to four settings at the time of the pilot-implementation, yet, we believe these provide us with a diverse experiences for the pilot study. These specific settings were chosen because these communities represent some of the largest ethnic groups in the Netherlands, and based on willingness of the community leader to co-organize the information sessions.

Based on earlier work [19], we co-organized the information sessions with people from the communities. This required establishing good rapport with community leaders, and discussions around the facilities needed to organize the session, and how we could ensure the session aligned with attendees’ cultural values. For instance, depending on community’s preferences, the information session had a more festive (West-African church and South-Asian Surinamese foundation) or more informative nature (both mosques). Moreover, together with the South-Asian Surinamese foundation, we co-created a play to address IHD symptoms and barriers to care, and simultaneously reduce the taboo on the topic of IHD. A few weeks before the information session, we trained the presenters, and provided them with slides, lessons plans, and additional information. Presenters were able to tailor the slides to community’s preferences and needs.

The information sessions consisted of two parts. First, there was a 45 min interactive presentation on the heart, (sex differences in) IHD symptoms (S3 File) and risk factors, and when to seek medical support. Slides were presented by a healthcare professional from a similar ethnic/cultural background, in the community’s native language. Second, with support from the Dutch Heart Foundation, we organized ‘checkpoints’ during these information sessions, as earlier work showed this would be a good motivator for people to attend an information session [19]. These checkpoints included free blood pressure-, blood glucose- and cholesterol- (both through finger prick), Body Mass Index-, and waist circumference measurements, along with personalized health advice. After the presentation and checkpoints, we shared informational flyers about IHD symptoms which were designed and tested with the target audience (S3 File).

### Evaluation methods and data collection

Lastly, the pilot-implementation of the toolkit was evaluated using a mixed-methods design, based on the RE-AIM framework (Table 2). Our quantitative evaluation included anonymous questionnaires (available in B1-level Dutch, English, or Turkish, S4 File) distributed amongst attendees of the information session, and data from the checkpoints, which were written down by trained volunteers performing the measurements. Our qualitative methods included observation forms (S5 File) filled out by 2–3 researchers during the information sessions, focus groups with attendees, and reflections with presenters, community leaders (influential figures within the community, e.g., the Imam and Pastor), and potential maintenance organizations (i.e., organizations in the social- and care domain that potentially have the structure and funding to maintain and scale the intervention). For readability, the evaluation strategies will be structured according to the RE-AIM framework.

**Table 2:** RE-AIM framework applied to the evaluation of a gender-informed and culturally-sensitive toolkit to promote symptom recognition and adequate care seeking in women and men across ethnic groups.

RE-AIM dimensions and the operational definitions	Indicators/nature of data	Measurement and sources of data
**Reach** refers to the absolute number, proportion (%), and representativeness of individuals who attended the sessions and participated in checkpoints	- Number and characteristics of attendees and individuals measured at checkpoints - Reasons for attending the information session	- Observation forms - Questionnaires - Checkpoint data - Focus groups
**Effectiveness** refers to the impact of the intervention on (short-term) outcomes	- Improvements in symptom recognition - Intention to change care seeking	- Questionnaires - Focus groups
**Adoption** refers to the absolute number, proportion (%), and representativeness of organizations willing to initiate and organize a session in their setting	- The willingness of organizations to co-create information sessions with us	- Reflection with community leaders
**Implementation** refers to the organizers’ and presenters’ fidelity to the various elements of the toolkit	- Was the toolkit implemented and used as intended	- Observation forms - Reflections with presenters
**Maintenance** refers to the extent to which the toolkit can be institutionalized in practice or policies, as well as sustainability over time and reinforcing factors	- What do organizations think about hosting and maintaining such a toolkit - Who could organize sessions in the future	- Reflection with potential maintenance organizations

#### Reach.

The reach of the intervention was evaluated using questionnaire, observation forms, and checkpoint data. Questionnaire data provided information on the number of attendees who filled in the questionnaire, and the age and sex of respondents. The number of attendees and the sex-distribution of attendees were also estimated in observation forms. Finally, check point data provided insights into the age, sex, and health parameters (blood pressure, blood glucose, cholesterol, Body Mass Index, and waist circumference) of people who had their measurements taken after the information session, including whether people previously knew they had high blood pressure/blood sugar/cholesterol (self-report). In addition, focus group data were used to understand motivations for attending the session.

#### Effectiveness.

The effectiveness was determined based on questionnaire and focus group data. The pre-test-post-test design of the questionnaire provided insights into people’s knowledge of IHD symptoms and risk factors before and after the presentation. Additionally, the questionnaire contained questions on satisfaction with the presentation on a scale using 5 smileys ranging from unhappy to happy, and a free space where respondents could provide feedback. Focus groups were conducted by [author4] and [author1] after the information sessions in all settings except the South-Asian Surinamese foundation; this was not possible due to the timing of the information session. In the mosques, separate focus groups were held for women and men due to cultural norms. We aimed to include six to eight women and six to eight men per setting, and potential participants were invited by asking if attendees were willing to participate in the focus group (i.e., convenience sampling). The topic list (S6 File) covered the following topics: general appraisal, knowledge improvement, intended changes in care seeking (i.e., what people would do when experiencing cardiac symptoms), appraisal of the various subcomponents (the information, presenter, slides, and checkpoints), and whether the organization and information aligned with their cultural norms and values. Participants were encouraged to freely share their opinions and perspectives. Moreover, an interactive component was incorporated in the focus groups to elicit a deeper discussion and reflection [21]. This involved participants expressing their preferences and opinions through voting with pawns on a range of images. Participants received a €25,- gift card as compensation for their time and effort. Focus group discussions were recorded using a digital audio recorder and lasted about one hour per focus group.

#### Adoption.

Data on the adoption were retrieved from reflections with community leaders, and covered topics such as general appraisal of the information session, motivations, conditions, and necessities for co-organizing the information session, promotion, agreements, success-factors, and points for improvement (S7 File). The evaluation with community leaders was conducted in a brief online survey for most communities. For one community, the community leader preferred having an informal online group discussion to discuss issues related to the adoption. This discussion lasted approximately 45 minutes, and while minutes were taken, the meeting was not audio recorded.

#### Implementation.

Reflections with presenters and observation forms were used to evaluate the implementation. In a brief online survey, presenters were asked about their general appraisal of the session, whether the materials were clear, and whether they had any tips for improving the material (S8 File). In the observation forms, researchers noted any infidelity to the intervention that was observed (i.e., when the intervention was not implemented as originally planned/intended), and whether individuals were paying attention, any disruptions, or other aspects that stood out during the implementation.

#### Maintenance.

Finally, findings concerning the maintenance of the toolkit were retrieved from reflections with potential maintenance organizations in the social or (health)care domain. These discussion took place online or live, and focused primarily on what these organizations would need for maintenance of the toolkit and continuation of the intervention (S9 File).

### Data analysis

With respect to the questionnaire data, knowledge improvement was determined by calculating the change in the proportion of respondents who correctly identified (sex differences in) IHD symptoms and risk factors before and after the presentation. Checkpoint data were analyzed by the Dutch Heart Foundation by calculating the number and proportions of elevated values. Data from observation forms and reflections with community leaders, presenters, and potential maintenance organizations were summarized and compared across settings. Finally, all focus group audio recordings were transcribed verbatim, anonymized, and analyzed using inductive coding and a thematic analysis by [author1], [author4], and [author2]. During data analysis, regular sessions were held amongst coders to discuss codes and emerging themes.

### Ethical considerations

The study was approved by the [institution] Medical Ethical Review Committee (reference W21_544 #21.600). Due to ethical and privacy regulation, we could not include people younger than 18 years old. Prior to enrollment, focus group participants provided written informed consent and oral approval to participate. Audio recordings were uploaded to the secured servers at [institution], in a folder only accessible by the lead researchers. Audio files and transcripts were transferred via a secured file transfer service. In line with [institution] privacy regulations, signed informed consent forms and paper surveys were saved in a secured cabinet at [institution]. Focus groups were anonymized during transcription, after which audio files were deleted. Interns who supported data collection signed a nondisclosure agreement. To limit the burden for participants with limited levels of literacy, information about the aim of the questionnaire and voluntary nature of participation was shared verbally prior to handing out the questionnaire. The questionnaire itself was anonymous, and participation in the session was not registered by name. Therefore, we did not collect formal informed consent forms for questionnaire participants. Ethical approval for the checkpoints was arranged by the Dutch Heart Foundation.

## Results

We successfully co-created and implemented a gender-informed and culturally-sensitive toolkit with the aim of promoting IHD symptom recognition and adequate care seeking. Below, we provide the results from the evaluation, structured around the RE-AIM framework.

### Reach

The number of attendees varied across data sources and settings. In total, there were 139 completed questionnaires (varying between seven to 46 across settings), 240 attendees according to observation forms (varying between 20–100 across settings) and 193 in checkpoint data (varying between 29–64 across settings). All data sources show that more women than men attended the information sessions (Tables 3 and 4). According to questionnaire data, 98 individuals (71%) who completed the questionnaire fell within the age range of 30–70, i.e., our target audience. Moreover, across data sources, the average age varied across settings, as the lowest average age (mean [standard deviation]) age found in the primarily West-African church (41.4 years [9.8]), versus the oldest age in the primarily South-Asian Surinamese mosque (68.0 years [7.5]). Among those measured at the checkpoints, we found relatively high numbers of elevated values, varying from 23% with high blood glucose to 70% with a large waist circumference (Table 5). Most attendees were reached by invitations from community leaders, advertisements on social media, and word-of-mouth advertising, and reasons for attending the information session included: 1) interest in/a need for information on the topic, 2) free health checks, and 3) habit, i.e., they frequently attend community gatherings.

**Table 3:** Questionnaire data on sociodemographic characteristics, attendees’ perceived ability to identify a heart attack, and attendees’ satisfaction.

	Total	Predominantly South-Asian Surinamese mosque	Predominantly West-African Church	Turkish Mosque	South-Asian Surinamese community
**Attendees (N)**	139	46	7	41	45
Women [%]	96 [69.1]	34 [73.9]	5 [71.4]	23 [56.1]	34 [75.6]
Mean age [SD]	62.7 [10.7]	68.0 [7.5]	41.4 [9.8]	59.4 [9.6]	63.8 [9.5]
Believes they are able to identify symptoms of a heart attack^a^ [%]	52 [37.4]	17 [37.0]	2 [28.6]	15 [36.6]	18 [40.0]
**Satisfaction** ^b^					
Overall, mean [SD]	4.61 [0.67]	4.59 [0.80]	5.0 [0.0]	4.66 [0.72]	4.52 [0.57]
Play, mean [SD]	–	–	–	–	4.36 [0.73]

SD, Standard Deviation. Data are presented as n [%] unless specified otherwise, and we included complete and incomplete cases. ^a^We recognize that a heart attack is not a synonym for IHD. However, to improve the readability of the questionnaire, we chose to use the term “heart attack” in the questionnaire, since this is a more familiar term and this term aligns with the appropriate ERK language level. ^b^A higher score equals greater satisfaction.

**Table 4:** The number and characteristics of people measured at the checkpoints.

	Total	Predominantly South-Asian Surinamese mosque	Predominantly West-African Church	Turkish Mosque	South-Asian Surinamese foundation
**Attendees**	193	46	29	64	54
Women	119 [61.7]	30 [65.2]	17 [58.6]	31 [48.4]	41 [75.9]
Age in years (mean)	56.5	64.4	44.5	52.9	64.3

Values are displayed as n or n [%], unless described otherwise. We included all individuals over the age of 18 years old, including individuals who did not have certain measurements taken.

**Table 5:** Results from checkpoint measurements.

	Total
**Systolic blood pressure (mmHg)**	185
<120	44 [24]
120-140	71 [38]
140-180	67 [36]
>180	3 [2]
*Newly-identified individuals with elevated values (≥140)*	14 [8]
**Total Cholesterol (mmol/L)**	141
<5.0	91 [65]
5.0-6.5	43 [31]
6.5-8.0	7 [5]
>8	0 [0]
*Newly-identified individuals with elevated values (≥5.0)*	16 [11]
**Blood Glucose (mmol/L)**	188
<7.8	144 [77]
7.8-11.1	31 [17]
>11.1	13 [7]
*Newly-identified individuals with elevated values (≥7.8)*	6 [3]
**Body Mass Index**	128
<18,5	21 [16]
18,5-25	30 [23]
25-30	51 [40]
>30	26 [20]
**Waist circumference in centimeters**	128
<80 (women) or <94 (men)	20 [16]
80-88 (women) or 94–102 (men)	21 [16]
>88 (women) or >102 (men)	87 [70]

All values are displayed as n, or n [%]. We included all individuals over the age of 18 years old, including individuals who did not have certain measurements taken.

*‘They announced it […]. The flyers were printed and we had to share them with the community. So yeah, I was in church that Sunday and the flyers were given to us, so I had to share it.’*

*- Woman from primarily West-African Church*

### Effectiveness

We found small short-term improvements in knowledge on (sex differences in) IHD symptoms and risk factors (Table 6). For instance, different IHD symptoms were correctly identified by 53.4–74.4% of participants in the pre-test, compared to 67.6–85.4% in the post-test. Similarly, numbers increased from 15.1–81.5% to 42.7–88.5% for different cardiovascular risk factors, and 30.5–60.2% to 52.2–75.0% for sex differences in IHD symptoms.

**Table 6:** Questionnaire data on respondents’ ability to identifying IHD symptoms and risk factors.

IHD symptoms	Pre	Post
Total n	133	103
Chest pain	99 [74.4]	88 [85.4]
Shortness of breath	74 [55.6]	69 [67.6]
Fatigue	71 [53.4]	70 [68.6]
Fever *(wrong answer)*	39 [29.3]	32 [31.1]
Pain when inhaling *(wrong answer)*	19 [14.3]	16 [15.5]
Pain radiating to the arms and jaw	71 [53.4]	74 [72.5]
All symptoms	1 [0.7]	2 [2.0]
Excluded due to missingness^a^	6	36
**Cardiovascular risk factors**		
Total n	119	97
Age	47 [39.5]	52 [54.2]
Sun exposure *(wrong answer)*	31 [26.1]	33 [34.0]
Gestational diabetes	18 [15.1]	41 [42.7]
Menopause	18 [15.1]	42 [43.8]
Hypertension	97 [81.5]	85 [88.5]
All risk factors	2 [1.7]	6 [6.4]
Excluded due to missingness^a^	20	42
**IHD symptoms more common in women**		
Total n	118	96
Pain radiating to the neck, back, shoulders, and stomach	62 [52.5]	72 [75.0]
Nausea	36 [30.5]	59 [64.1]
Fainting	39 [33.1]	48 [52.2]
Headache *(wrong answer)*	21 [17.8]	16 [16.7]
Heart palpitations	71 [60.2]	60 [65.2]
All symptoms	1 [0.8]	2 [2.2]
Excluded due to missingness^a^	21	43
**What to do when experiencing IHD symptoms in rest**		
Total n	125	93
Call emergency number 112 *(correct answer)*	60 [48.0]	53 [56.4]
Contact GP	19 [15.1]	14 [14.6]
Contact family	6 [4.8]	5 [5.3]
Bring to hospital	1 [0.8]	0 [0.0]
Wait it out	2 [1.6]	2 [2.1]
I don’t know	6 [4.8]	0 [0.0]
More options selected^b^	31 [24.6]	19 [20.4]
Excluded due to missingness^a^	14	46
**What to do when experiencing IHD symptoms during exertion**		
Total n	127	93
Call emergency number 112	57 [44.9]	35 [37.2]
Contact GP *(correct answer)*	21 [16.5]	25 [26.6]
Contact family	5 [3.9]	4 [4.3]
Bring to hospital	0 [0.0]	1 [1.1]
Wait it out	10 [7.9]	5 [5.3]
I don’t know	5 [3.9]	0 [0.0]
More options selected^b^	29 [22.8]	23 [24.7]
Excluded due to missingness^a^	12	46

All data are presented as n [%], and only complete cases were included. For the questions on symptoms and risk factors, these are numbers of people who answered these questions correctly. ^a^Percentages were calculated excluding individuals who did not select any answer option, as they were assumed to have skipped this question. Greater drop-out at the post-test versus pre-test has resulted in lower absolute numbers of correct answers, yet higher relative proportions of correct answers for some questions. ^b^Despite being instructed to select one answer, several people selected multiple options.

We organized five focus groups across three settings, with a total of 32 participants (19 women and 13 men). Overall, participants said that they found the session informative, and that they learned new information about IHD symptoms, risk factors, and in particular sex differences in IHD symptoms. For instance, when asked if participants were previously aware of sex differences in IHD symptoms and risk factors, one woman stated:

*“For me, no. First time. And I was surprised as well because I didn’t think menopause would have anything to do with a heart attack.”*

*- Woman from primarily West-African Church*

Additionally, most participants felt confident in their ability to recognize a heart attack after the session. Some participants stated they would have liked to receive more in-depth information, however, this mostly concerned people diagnosed with IHD, who were not the target audience. Focus group participants also said the effectiveness could be improved by increasing the length of the presentation, making it more visual, and through repetition of the message both during the presentation and over time.

*‘It’s a one-off, yes, all information is given at once and you will only remember certain things. […] Repetition would be better for us. Also because most of the seniors are 65 + . They don’t remember things as quickly and so that’s why repetition is important. ‘*

- *Man from predominantly South-Asian Surinamese mosque*

### Adoption

We planned to organize four information sessions in our pilot-implementation study. In total, we contacted five communities with the request to co-organize an information session; one community, however, was unable to host a session due to other priorities. Nevertheless, this shows a high level of adoption amongst the contacted communities, which is further exemplified by additional requests for information sessions in other communities after the pilot-implementation study.

Reflections with community leaders’ revealed that their main motivation for collaborating and hosting such an information session was the high level of interest in heart disease. They often recognized IHD as a large issue in their community, and they felt that their organization had a responsibility in sharing information. Other motivations for hosting an information session included the co-creative approach taken in the process, and the pleasant collaboration between community leaders and researchers. In some communities, it was necessary to visit the community several times to develop trust with community leaders and members, in particular communities that experienced more mistrust towards Dutch institutions. Across settings, an important facilitator for implementation was the prior experience in organizing community events by the community. Challenges to the adoption mostly related to a lack of clear communication concerning finances and planning of the information sessions, as discussing expectations and setting appointments was deemed important to prevent miscommunication.

### Implementation

The implementation of the intervention varied somewhat across settings, given that the intervention components were tailored to community preferences, including, e.g., the degree of festivity of the information session. Beyond the tailored intervention components, we observed several instances of infidelity to the implementation. For instance, while the ability to adjust slides to the audience was considered a strength, one presenter took out the interactive components, while interactive and narrative information was used to tailor the information to different cultural backgrounds. Focus group participants in this community said that the lack of interaction led to lower satisfaction, and participants from other communities stated that interactive components such as the anecdotal patient story, were very important and impressive:

*“[The anecdotal patient story], was a good example […]. First, she has a daughter, and then comes a divorce, and after that comes the grieve, and before she even grieved, she has a heart attack”*

*- Woman from Turkish mosque*

Moreover, during the final rehearsal of the play, it appeared that the scriptwriters from the community had altered the description of IHD symptoms, which was no longer medically accurate. One final point concerning fidelity was related to the presenter, as one of the presenters was not a medical doctor. This presenter did not possess enough medical knowledge to answer all the questions from the audience, which led to lower satisfaction.

*‘He’s not a doctor, […] and he has very little knowledge about the heart. Maybe he prepared well in advance, but not enough to be able to answer all questions.’*

*- Man from Turkish mosque*

Other points of improvement during the implementation were related to the organization of the session, including organizational and technical difficulties that hindered the information sessions. This included disruptions during the presentation when food, drinks, or health checks were offered during the presentation, and issues with the internet connection, microphones malfunctioning, and the visibility of the screen for those seated in the back. Finally, while women and men were seated separately in both mosques, women in one mosque said they still felt uncomfortable sharing personal questions regarding, e.g., menopause in the presence of men.

*‘Yes, the doctor asked about menopause, but you can’t say that right in front of the men. I wanted to respond, but the men are there, so you can’t say that publicly. So if it was just a group of women, we could tell you more.’*

*- Woman from Turkish mosque*

### Maintenance

Challenges and opportunities for maintenance of the toolkit were discussed with several potential maintenance organizations. Two main prerequisites for maintenance of the toolkit and continuation of the intervention emerged: 1) experience with, and existing infrastructure for, organizing community events, and 2) one individual responsible for the implementation and maintenance. This person in charge requires sufficient training in using the toolkit to promote fidelity in the adoption of the toolkit. While all organizations expressed high levels of interest in the intervention, the willingness to use the intervention was lower in organizations that had no experience in organizing community events. Other opportunities for maintenance included offering the toolkit as an addition to pre-existing activities within organizations, as well as promoting the toolkit amongst communities in order for them to organize information sessions self-sufficiently.

The maintenance of the intervention was discussed with several parties during the study. The intervention has since been hosted by Municipal Health Services Haaglanden. This organization works with community health ambassadors, who have a large network within different ethnic communities, and organize various different activities in the area of health promotion. These health ambassadors were instructed how to use the toolkit and share the information within different communities, including which information could be adapted to fit communities’ preferences and needs, and which information encompassed core elements and should not be changed.

## Discussion

In this study a gender-informed and culturally-sensitive toolkit to improve IHD symptom recognition and promote adequate care seeking was developed and pilot-implemented. In the evaluation, we found high levels of satisfaction among attendees, and a small (short-term) increase in knowledge on IHD symptoms and risk factors. Nevertheless, in terms of implementation in line with the RE-AIM framework, challenges regarding the adoption and maintenance of the toolkit remained.

### Limitations

Several limitations to this work should be considered. One main limitation pertains to the data collection methods, including the questionnaire and focus group discussions. First, there were large discrepancies in the number of attendees, people measured, and completed questionnaires. This can partially be explained by the presence of minors in some settings (specifically the West-African church), but may particularly reflect a low, potentially selective willingness to fill in the questionnaire. Beyond the small sample size, a large number of participants did not complete the post-test questionnaire, resulting in large numbers of excluded participants and lower reliability of the questionnaire data. Therefore, while questionnaire results are consistent with focus group findings on small, short-term knowledge improvements, the questionnaire data should be interpreted cautiously, especially given that questionnaire drop-out may be selective, and those who dropped out at the post-test may not have participated actively during the presentation. This further suggests that for future evaluations, other evaluation strategies may be considered, including more interactive strategies that do not require literacy, such as actor depicting symptoms and verbally discussing what people the right action would be. Second, social desirability bias could have affected people’s answers during focus group discussions [22]. To mitigate this, we explicitly asked participants to answer honestly, and applied data triangulation by interviewing a diverse range of participants and professionals, which provided a broad range of perspectives on the topic. Finally, evaluation results concerning, e.g., increased knowledge on IHD, are limited to the short-term, and do not provide insights into long-term effectiveness. The long-term effectiveness of the intervention is an interesting direction for future research, also given the lack of research on the long-term effects of one-time interventions on knowledge improvement.

### Reach

Despite some variations across settings, the interventions achieved a relatively large reach. Women and somewhat older individuals appeared to be slightly overrepresented, which may reflect the typical composition of these community organizations, or may signal a greater interest in heart disease among these groups. The presence of individuals with IHD was largely unknown, with the exception of some focus group participants who disclosed their condition. However, the intervention appeared to successfully reach the target audience based on age and risk factors. Previous research suggests [23], that defining a target audience should consider not only the number of people and the proportion of at-risk individuals, but also practical aspects such as resources and the persuasibility and accessibility of the population, as well as ethical considerations such as equity. Thus, to further specify the target audience, it is important to take into account that IHD risk may present earlier in certain high-risk ethnic groups [24,25], and therefore, certain populations may be targeted at a younger age. Clearly defining and effectively communicating the target audience, while prioritizing their reach, could enhance the intervention’s overall effectiveness.

### Effectiveness

The results with respect to effectiveness suggest that the toolkit led to small, short-term improvements in knowledge on (sex differences in) IHD symptoms and cardiovascular risk factors, however, the high drop-out rate may have significantly affected the reliability of results. Moreover, the long-term effectiveness also remains uncertain, as such knowledge improvements may diminish over time [26]. To mitigate this, informational flyers and videos were distributed to reinforce the key information and support retention [19]. In line with previous research [27], focus group findings indicate that spaced message repetition is crucial for sustained symptom recognition. While repetition during the presentation is easily implemented, maintaining it over time may be more challenging and costly, requiring the organization of frequent community gatherings. Alternatively, instead of frequent repetition of the message, the solution may be sought in an easy-to-remember message or a mnemonic device [28]. A well-known example is the stroke awareness campaign F.A.S.T. (Face, Arm, Speech, Time), a multi-phase campaign that included television, press, and radio advertisements presenting a mnemonic device to promote recognition of stroke symptoms. This campaign was proven effective in promoting stroke symptom recognition and care seeking for stroke symptoms on the long term [29]. A similar mnemonic device might improve IHD symptom recognition. However, unlike stroke, which has a distinct symptom profile, IHD presents with more varied and less specific symptoms, making a direct translation of this approach less straightforward.

Beyond the effect on knowledge of IHD symptoms, the effect on care seeking behavior should be critically assessed. Increased knowledge of IHD symptoms and when to seek care could reduce the number of missed or delayed IHD diagnoses. However, referring back to the causal chain described under ‘intervention components’, we were only able to empirically assess short-term knowledge changes and intended care seeking, yet we were unable to assess the effect on actual care seeking. Moreover, unwanted side effects may occur, and should be assessed. For instance, given the various nature of potentially cardiac symptoms, and the high prevalence of these symptoms in the general population [30,31], large-scale information campaigns may spike the number of (unnecessary) healthcare visits for these symptoms [19]. This could pose a burden for the healthcare system, especially given the already high workload in Dutch general practice care [32].

### Adoption

While we were unable to quantify findings on the adoption, we observed a high adoption rate of the intervention among the approached communities, partly due to interest in the topic of IHD. However, strong rapport between community leaders and researchers also played a key role, serving both as a motivation, and in some cases, a starting point for collaboration. This relates to the ethics of co-creation, referring to the invisible work done by participatory researchers around the ethical aspects of collaborating with patients or participants [33]. In particular, in communities that expressed more mistrust towards Dutch institutions [34], emotion- and relationship work was necessary to foster the necessary trust for collaboration [35]. This also required awareness of unequal power dynamics, and acknowledging how societal and systemic issues led to this mistrust. While preventing unequal power dynamics may be difficult in practice, there is significant attention for promoting equity in co-creation within the implementation science literature [36–38]. Literature describes various different models, frameworks, and theories for implementation scientists studying ethnicity-related health inequalities [37], and experts in this field created a charter which includes seven principles to promote equity in co-creation [36]. Our experiences align with these studies, as we found fostering trust, intentionally including a diverse range of individuals in the co-creation process, acknowledging ongoing and historical harms [36], overcoming implicit bias, and reflecting on one’s own positionality [38] were important prerequisites to promote equity in co-creation.

Moreover, in line with previous research, community engagement [39,40] and cultural-sensitivity of the intervention [40,41] were important facilitators for the adoption and effectiveness of the intervention. Attendees and community leaders specifically mentioned the co-creative approach taken during the development and implementation, the visual, narrative, interactive, and easy-to-understand presentation, the knowledgeable speaker with a similar cultural or ethnic background, and the free health checks. Overall, this led to high levels of satisfaction across all settings, while the acceptability of interventions is known to vary across cultural groups [42]. This suggests that the adaptability of the intervention may enhance both acceptability and social validity of the intervention across different settings [42].

### Implementation

With respect to the implementation, facilitators included an interest in the topic of heart disease, and good rapport with community leaders and members for the collaboration. This good report and the collaboration with communities may be a double-edged sword, as this allowed us to share the organizational work with them, and it led to improved satisfaction and ownership amongst communities. One notable challenge to the implementation was infidelity to the intervention. While the adaptability of the toolkit was considered a great strength, including adapting the language and the content of the presentation to attendees’ cultural background [43], this adaptability may negatively affect fidelity to the intervention when intervention components that should not be adapted are altered (i.e., drift) [44], and could thus have affected the effectiveness of the intervention. Therefore, the fidelity to the toolkit should be safeguarded, to ensure that essential intervention components, in particular the medical information and presence of interactive elements, are not altered or excluded.

Other challenges to the implementation were related to organizational challenges, and a lack of clear communication about planning and finances. To prevent these challenges in future information sessions, we have now incorporated recommendations to improve the organization of sessions. This includes recommendations for clear communication (such as discussing finances and dividing responsibilities), and more practical recommendations (e.g., checking if the technological equipment is working, a checklist of all necessary resources) in the toolkit. Nevertheless, it is important to recognize that implementation of interventions in real-world settings means there is no absolute control over the circumstances, and therefore, some degree of flexibility on the organization’s behalf is required. However, a strength related to the development and implementation of this toolkit in real-world settings instead of experimental settings, means that the generalizability of the intervention can be considered high [45].

### Maintenance

Despite the significant attention for early recognition of IHD at an (inter)national [46,47] level, and the high demand from communities for information sessions, the maintenance of the toolkit appeared to be challenging, as this required the integration of the intervention into existing infrastructure within a potential maintenance organization [42]. This required financial and material (e.g., for the rent of locations, audiovisual media, flyers) as well as human resources (presenters, organizers) for the organization of such live sessions. While budget was available within this study, finding and securing long-term funding for such activities is challenging. Moreover, maintenance of the intervention required experience with setting up such live community sessions and a large network among different ethnic minority groups. This is highly dependent on the network, experience, and organizational skills and capacity of the person in charge of organizing these sessions. This reliance on individual networks, skills, and experience may harm the sustainability and broader implementation of the intervention. We found these resources and this experience within the Municipal Health Services Haaglanden, the organization currently in charge of maintenance and scaling of the toolkit. Finally, in addition to determining the effectiveness, for potential maintenance organizations it would also be relevant to determine the costs associated with the implementation and maintenance, and these should be weighed against the expected effects of the intervention.

### Conclusion

In conclusion, we were able to successfully co-create and pilot-implement a toolkit to promote IHD symptom recognition. The toolkit was well-received by attendees and community leaders, and findings concerning the reach, adoption and implementation were positive. The (cost)effectiveness should be further evaluated to study the long-term effect on symptom recognition, as well as the effect on care seeking. Moreover, the need for integration of the intervention in current infrastructure constitutes a challenge to the maintenance of the toolkit.

## Supporting information

S1 File Background intervention development. (DOCX)

S2 File Logic model. (DOCX)

S3 File Background video and flyer development. (DOCX)

S4 File Pre- and post-test questionnaire. (PDF)

S5 File Observation forms. (DOCX)

S6 File Topic list focus groups. (DOCX)

S7 File Reflection and evaluation organizers. (DOCX)

S8 File Reflection and evaluation with presenters. (DOCX)

S9 File Topic list for discussions with maintenance organizations. (DOCX)

S10 File Deidentified questionnaire data. (XLSX)
