# Supplementary material for: Implementation and product- and process evaluation of a co-created gender-informed and culturally-sensitive toolkit to improve symptom recognition and care seeking for ischemic heart disease: RE-AIM framework
Source: PLoS One. 2026 Mar 5;21(3):e0344093. doi: 10.1371/journal.pone.0344093 (PMC12962543; doi:10.1371/journal.pone.0344093)
Supplement: S1 File — (DOCX) [file pone.0344093.s001.docx]

**Intervention development**

In co-creation with citizens, patients, community leaders, and medical professionals from different ethnic groups, we developed the gender- and culturally-sensitive toolkit to improve IHD symptom recognition and reduce barriers to care. The development consisted of several feedback rounds from co-creation partners, at different steps of the development process. Based on the qualitative work on HCSB and health information preferences, we developed initial ideas on what tools were needed within the toolkit. We first asked for feedback from community leaders, to make sure the toolkit was complete and aligned with their cultural values and norms: as suggested by one of the community leaders, we added in the script for the theatre play. Next, we developed prototypes for the informational flyer, video’s, and the slides to be used during the presentation. First, we asked for feedback from a medical perspective from a group of experts in the field of general practice. Next, we showed these materials to a group of citizens from different ethnic groups, to ensure that the materials were comprehensible for people with different cultural backgrounds and varying levels of Dutch language literacy, and to check that the translations of the materials were done correctly. Finally, the reflections with potential maintenance organizations were used to gain insights on what else was needed to be able to maintain and carry out the intervention. Feedback was incorporated at different rounds, and the final evaluation took place after the pilot-implementation.

Based on participants’ preferences, the content of the intervention, as well as the design of the intervention, were tailored to gender and ethnicity. With respect to the content of the intervention, this was tailored to gender by incorporating sex differences in IHD symptom presentations and sex-specific risk factors. Ethnicity was incorporated by including ethnic differences in IHD risk profiles and risk factors, as well as religious and cultural factors that may affect care seeking. With respect to the design of the intervention, this was tailored to gender and ethnicity by e.g. tailoring the location, separating women and men when necessary, and having a presenter who is similar to the audience.

The final toolkit includes various ‘tools’ to help professionals and organizations reach women and men of different ethnic backgrounds in the Netherlands, inform them about IHD symptoms, and promote adequate care seeking. The target audience of this intervention is primarily women and men from different ethnic backgrounds, between the ages of 30-70, who may be at risk of IHD, without a pre-existing IHD diagnosis. While the toolkit was developed with input from individuals of native Dutch, South-Asian Surinamese, African Surinamese, Ghanaian, Turkish, and Moroccan ethnic origin, the toolkit may be tailored to the preferences and needs of individuals from different ethnic groups as well. Since the barriers to care and information preferences and needs were mostly comparable across different ethnic minority groups[17,19], these may also relate to other ethnic groups than those included in these studies. Therefore, slight alterations to the intervention (e.g. changing the language of the presentation or the description of the introductory anecdotal story, but not the medical information), may allow this intervention to be implemented in other communities as well.
