# Supplementary material for: Implementation and product- and process evaluation of a co-created gender-informed and culturally-sensitive toolkit to improve symptom recognition and care seeking for ischemic heart disease: RE-AIM framework
Source: PLoS One. 2026 Mar 5;21(3):e0344093. doi: 10.1371/journal.pone.0344093 (PMC12962543; doi:10.1371/journal.pone.0344093)
Supplement: S2 File — (DOCX) [file pone.0344093.s002.docx]

| **Inputs** |  | **Activities** |  | **Outputs** |  | **Short term outcomes** |  | **Intermediate outcomes** |  | **Impact** | |
| --- | --- | --- | --- | --- | --- | --- | --- | --- | --- | --- | --- |
| - Financial resources - Time - Human capital - Network within communities - Partnerships with professional organizations - Expertise - Equipment for live information sessions and materials | 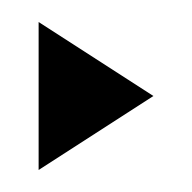 | - Training for (community) health educators - Creation of toolkit and informative materials which can be spread in communities | 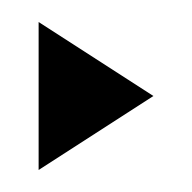 | A toolkit including tools for   - Live information sessions - Video’s - Flyers | 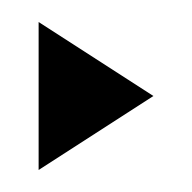 | - Improved IHD symptom recognition among women and men of different ethnic groups in the Netherlands | 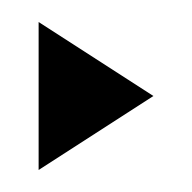 | - Improved care seeking for symptoms suggestive of IHD - Fewer delays in care seeking for symptoms suggestive of IHD | 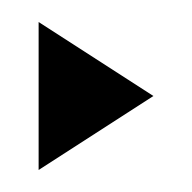 | - Improved health outcomes for women and men of different ethnic groups - Reduction in ethnic health disparities | |
|  | | | | |  |  | | | | |  |
| **Assumptions**   - The manner in which information is offered will ensure that people will attend the live information sessions - When attending these live information sessions, individuals will pay attention and retain information - More knowledge on IHD symptoms ensures that people will recognize symptoms when they are experiencing them - Seeking care in a timely manner will improve care and health outcomes for patients | | | | |  | **External factors**   - Prioritization of (reducing) cardiovascular burden of disease - Prioritization of reducing ethnic health disparities - Funding within the adopter of the intervention | | | | |  |
