# Supplementary material for: Implementation and product- and process evaluation of a co-created gender-informed and culturally-sensitive toolkit to improve symptom recognition and care seeking for ischemic heart disease: RE-AIM framework
Source: PLoS One. 2026 Mar 5;21(3):e0344093. doi: 10.1371/journal.pone.0344093 (PMC12962543; doi:10.1371/journal.pone.0344093)
Supplement: S3 File — (DOCX) [file pone.0344093.s003.docx]

**Development of flyers and videos to promote symptom recognition**

We developed informational flyers and videos to promote symptom recognition for acute- and non-acute IHD. These flyers were co-created with input from patients, citizens, community leaders, and various healthcare professionals. The flyers and videos showed images of ethnically-diverse women and men exhibiting typical cardiac symptoms. The symptom presentations were based on current Dutch general practice and cardiology guidelines, as well as discussions with experts in the field. For acute IHD, these symptoms were chest pain and pain radiating to the arms and jaw, especially in the presence of nausea and excessive sweating. For non-acute events, these were chest pain and pain radiating to the arms and jaw, especially in the presence of shortness of breath, severe fatigue elicited by physical exertion, palpitations, dizziness or fainting, and pain that spreads to the neck, back, shoulders, and stomach. For acute IHD, people were advised to call emergency services, and for non-acute IHD they were advised to visit their GP. Materials were developed in Dutch, English, Turkish, and Arabic.

To test our informational videos and flyers, user tests were conducted with lower SES Dutch, Turkish, Moroccan, and African individuals. User tests for flyers were conducted using a standardized method(45) consisting of four steps: first, the images or pictures were discussed to study how participants interpreted these without text. Second, participants were asked to read the text aloud to identify any difficult words or unclear texts. Third, participants were asked to describe the message of the flyer in their own words, and fourth, we asked participants for advice to make the material clearer or easier to understand. In some instances, due to limited concentration, the summary part and advice for improvement were omitted. For the user tests of the videos, people were also asked whether images and text were clear, and also whether the timing of images and text were synchronized.

During the user tests, notes were taken to summarize findings and identify tips to improve the materials. These notes were compiled into one document per image. For each image in the information folder, we evaluated whether any changes were needed to improve the clarity of the information. These changes were implemented prior to use in practice. The same procedure was followed for the videos.
