# Supplementary material for: Implementation and product- and process evaluation of a co-created gender-informed and culturally-sensitive toolkit to improve symptom recognition and care seeking for ischemic heart disease: RE-AIM framework
Source: PLoS One. 2026 Mar 5;21(3):e0344093. doi: 10.1371/journal.pone.0344093 (PMC12962543; doi:10.1371/journal.pone.0344093)
Supplement: S4 File — (PDF) [file pone.0344093.s004.pdf]

Before the presentation

We want to know what you already know about a heart attack. This questionnaire will take no more than 5 minutes. You may answer as honestly as possible and the questionnaire is anonymous.

1. I am a: O Woman O Man O Other O Rather not say

2. I am ________ years old

3. I recognize the symptoms of a heart attack: O No O Yes

4. Which symptoms are associated with a heart attack?

	NO	YES	I DON'T KNOW
Chest pain or pressure	O	O	O
Pain when breathing in	O	O	O
Radiating pain to jaw and arms	O	O	O
Rapid breathing and/or feeling short of breath	O	O	O
Fever	O	O	O
Fatigue	O	O	O

5. Who has a higher risk of having a heart attack?

	NO	YES	I DON'T KNOW
Older people	O	O	O
People who get a lot of sun exposure	O	O	O
Women with diabetes during pregnancy	O	O	O
Women after menopause	O	O	O
People with high blood pressure	O	O	O

6. Which symptoms are more common in women and the elderly than in men?

	NO	YES	I DON'T KNOW
Pain radiating to neck, back, shoulders or abdomen	O	O	O
Feeling nauseous	O	O	O
Fainting and/or dizziness	O	O	O
Headache	O	O	O
Your heart is beating very fast	O	O	O

7. What do you do when someone experiences heart symptoms **during physical activity** (for example walking, vacuuming and climbing stairs)? ***Choose 1 answer)***

O Wait for it to pass
O Call the GP/family doctor
O Call 112 (emergency number)
O Call family for advice
O Take to hospital
O I don't know

8. What do you do when someone has heart symptoms **at rest** that do not stop ***(Choose 1 answer)***

O Wait for it to pass
O Call the GP/family doctor
O Call 112 (emergency number)
O Call family for advice
O Take to hospital
O I don't know

Fill in the back after the presentation

After the presentation

about a heart attack? You may answer as honestly as possible and the questionnaire is anonymous.

1. Which symptoms are associated with a heart attack?

	NO	YES	I DON'T KNOW
Chest pain or pressure	O	O	O
Pain when breathing in	O	O	O
Radiating pain to jaw and arms	O	O	O
Rapid breathing and/or feeling short of breath	O	O	O
Fever	O	O	O
Fatigue	O	O	O

2. Who has a higher risk of having a heart attack?

	NO	YES	I DON'T KNOW
Older people	O	O	O
People who get a lot of sun exposure	O	O	O
Women with diabetes during pregnancy	O	O	O
Women after menopause	O	O	O
People with high blood pressure	O	O	O

3. Which symptoms are more common in women and the elderly than in men?

	NO	YES	I DON'T KNOW
Pain radiating to neck, back, shoulders or abdomen	O	O	O
Feeling nauseous	O	O	O
Fainting and/or dizziness	O	O	O
Headache	O	O	O
Your heart is beating very fast	O	O	O

4. What do you do when someone experiences heart symptoms **during physical activity** (for example walking, vacuuming and climbing stairs)? ***(Choose 1 answer)***

O Wait for it to pass
O Call the GP/family doctor
O Call 112 (emergency number)
O Call family for advice
O Take to hospital
O I don't know

5. What do you do when someone has heart symptoms **at rest** that do not stop? ***(Choose 1 answer)***

O Wait for it to pass
O Call the GP/family doctor
O Call 112 (emergency number)
O Call family for advice
O Take to hospital
O I don't know

6. What do you think of this meeting?

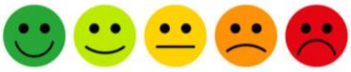

O O O O O

7. Do you have any tips to improve this meeting?

____

____

Thank you very much for completing the questionnaire!
