# Supplementary material for: Implementation and product- and process evaluation of a co-created gender-informed and culturally-sensitive toolkit to improve symptom recognition and care seeking for ischemic heart disease: RE-AIM framework
Source: PLoS One. 2026 Mar 5;21(3):e0344093. doi: 10.1371/journal.pone.0344093 (PMC12962543; doi:10.1371/journal.pone.0344093)
Supplement: S5 File — (DOCX) [file pone.0344093.s005.docx]

**Observation forms filled in during the presentations.**

Observations

*Observer: Location:*

How many people are there?

Is everyone there right away, do people come in later, or leave early?

Is one actively listening and paying attention?

Are people talking or doing other things during the presentation?

Is the location/room/space suitable, can everyone see and hear everything well?

Do people ask questions during/after the presentation? (disruptive during session?)

What questions do people ask?

Do people actively participate in the interactive parts of the presentation?

Do people discuss the information session? After the presentation?
