# Supplementary material for: Implementation and product- and process evaluation of a co-created gender-informed and culturally-sensitive toolkit to improve symptom recognition and care seeking for ischemic heart disease: RE-AIM framework
Source: PLoS One. 2026 Mar 5;21(3):e0344093. doi: 10.1371/journal.pone.0344093 (PMC12962543; doi:10.1371/journal.pone.0344093)
Supplement: S6 File — (DOCX) [file pone.0344093.s006.docx]

**English topic list used during focus groups.**

| *Topic* | Questions and (possible) probing questions |
| --- | --- |
| General & improvements  (Using Images with smileys) | Why did you come to this session?  How did you know about this session?  How do you feel about this session?  What did you learn?  What do you like or dislike about the session? |
| Knowledge/symptom recognition: You just learned what a heart attack is, and what symptoms people may experience during a heart attack. (Using images with symptoms)  Woman/Man differences: We also talked about the differences in symptoms in women and men. | Did you learn something about these symptoms you didn't already know?  How confident do you feel now about recognizing the symptoms of a heart attack?  What did you already know about differences in symptoms between women and men? What have you learned new? |
| Behavioral (intent): We talked about the barriers that cause people not to go to the doctor or to go too late. Lack of knowledge is one cause of this. We also talked about what action to take in the event of a heart attack. (Using images with barriers to seeking healthcare) | Would you go to the doctor/GP now with these symptoms? What else would you need? Why?  What obstacles are you still facing?  What can we do about these obstacles? |
| Organization: We just had a presentation, you can have measurements done by the Dutch Heart Foundation, and we have flyers. We would like to know what you thought of these components.  (Using images with smileys)  We have also talked about differences in women and men. | What did you think of the different components of the session?  Information  Presentation  Speaker  Measuring points  The wishes of women/men? (form, obstacles to seeking care)  How does the session connect to/fit with your community? |
| Other: (Give summary in advance of the mentioned good points and improvements) | What stuck out to you the most?  How can we motivate people to come to such a session? |
| Closing: Thank you for your time and participating in this group discussion. | Are there any topics for you that we have not discussed that you would like to share? |
