# Supplementary material for: Implementation and product- and process evaluation of a co-created gender-informed and culturally-sensitive toolkit to improve symptom recognition and care seeking for ischemic heart disease: RE-AIM framework
Source: PLoS One. 2026 Mar 5;21(3):e0344093. doi: 10.1371/journal.pone.0344093 (PMC12962543; doi:10.1371/journal.pone.0344093)
Supplement: S7 File — (DOCX) [file pone.0344093.s007.docx]

**Reflection and evaluation questions organizers**

General impression of the information session

- Overall, how do you reflect on the information session?
- Was the information session well prepared? Why or why not?
- How did the information session go? Were there unexpected problems during the information session, or positive surprises?

Motivation for collaborating:

- Why did you want to organize this information session with us?
- What were the most important reasons or goals for you to support this information session?

Conditions and necessities:

- What were the most important condition that allowed you to organize this information session?
- What did you do to organize this information session? Which steps did you and the committee take?
- What did you need for the organization? Was something missing, or did you expect more support from our team?

Promotion:

- How did you promote the information session amongst people?
- Which methods or channels did you use to boost the information session?

Organization and agreements:

- Which agreements were made about the organization of the information session (how were tasks and costs divided)?
- Were the agreements made clear and feasible? Why or why not?

Important success-factors:

- Which factors are important for the success of the information session (e.g. promotion, financial means, checkpoints, connections, catering, the presence of community leaders)?
- Why do you think these factors were crucial for the success of the information session?
- What went well, and should we definitely do again in the future?

Points of improvement:

- What could have gone better in organizing the information session? (e.g. timing, location, facilities, presenters, checkpoints and materials, sufficient staff)?
- Which specific things were missing, or could have been arranged better?

Future:

- If, in the future, you would host another information sessions: what would you need from us to organize this information sessions self-sufficiently?
