# Supplementary material for: Implementation and product- and process evaluation of a co-created gender-informed and culturally-sensitive toolkit to improve symptom recognition and care seeking for ischemic heart disease: RE-AIM framework
Source: PLoS One. 2026 Mar 5;21(3):e0344093. doi: 10.1371/journal.pone.0344093 (PMC12962543; doi:10.1371/journal.pone.0344093)
Supplement: S8 File — (DOCX) [file pone.0344093.s008.docx]

**Reflection and evaluation presenters**

- Looking back, how did the presentation go? What went well, what could have gone better?
- Do you have any tips for the presentation or the materials?
- Was the material clear (the lesson plan, the introductory anecdote, the information about barriers to care)?
- Was it necessary to go through the presentation together, or could you have done this using only the materials?
- Any other things that stood out to you, or that you want to mention?
