# Supplementary material for: Implementation and product- and process evaluation of a co-created gender-informed and culturally-sensitive toolkit to improve symptom recognition and care seeking for ischemic heart disease: RE-AIM framework
Source: PLoS One. 2026 Mar 5;21(3):e0344093. doi: 10.1371/journal.pone.0344093 (PMC12962543; doi:10.1371/journal.pone.0344093)
Supplement: S9 File — (DOCX) [file pone.0344093.s009.docx]

**Topic list potential maintenance organizations**

Brief introduction of the toolkit and the implementation process so far

- What does [organization] need for successful adoption of this toolkit?
- Are the toolkit and usage of the toolkit self-evident?
- How can [organization] use and implement this toolkit?
- Who/what department would be responsible?
- Collaboration between different parties?
- Who should know about this toolkit?
- Organization of sessions: [organization] or host organization?
- Offer toolkit in addition to other services?
- Findable toolkit: where/how could [organization] host the toolkit?
- How to go about updating information
- What does [organization] need for the broader implementation of the toolkit?
- Manpower and network
- Finances
- Internal promotion
- Presentation for employees
- Via which channels/organizations can we promote the toolkit?
- External promotion?
- What are barriers and facilitators to the adoption and broader implementation?
